# Promoting exercise training and physical activity in daily life: a feasibility study of a virtual group intervention for behaviour change in COPD

**DOI:** 10.1186/s12911-018-0721-8

**Published:** 2018-12-18

**Authors:** Tatjana M. Burkow, Lars K. Vognild, Elin Johnsen, Astrid Bratvold, Marijke Jongsma Risberg

**Affiliations:** 1 University Hospital of North Norway, P.O. Box 35, N-9038 Tromsø, Norway; 2 Norut, P.O. Box 6434 Forskningsparken, N-9294, Tromsø, Norway

**Keywords:** Behaviour change intervention, Virtual group, COPD, Physical activity, Exercise training, Behaviour change technologies, Application (app), Tablet computer, Gamification, Self-monitoring of behaviour

## Abstract

**Background:**

Physical inactivity is associated with poor health outcomes in chronic obstructive pulmonary disease (COPD)**.** It is therefore crucial for patients to have a physically active lifestyle. The aims of this feasibility study were to assess a tablet-based physical activity behavioural intervention in virtual groups for COPD regarding 1) patients’ acceptance 2) technology usability 3) patients’ exercise programme adherence and 4) changes in patients’ physical activity level.

**Methods:**

We used an application with functionality for a virtual peer group, a digital exercise diary, a follow-along exercise video, and visual rewards on the home screen wallpaper. The exercise programme combined scheduled virtual group exercising (outdoor ground walking, indoor resistance and strength training) with self-chosen individual exercises. Ten participants with COPD were enrolled into two exercise training groups. Patients’ acceptance was assessed by semi-structured interviews, technology usability was assessed by the System Usability Scale, and exercise programme adherence and level of physical activity by self-reporting. The interviews were also used for the latter three aspects.

**Results:**

The virtual peer group was experienced as motivating, helping participants to get started and be physically active. They updated their own activity status and kept track of the others’ status. Having a time schedule for the virtual group exercises helped them to avoid postponing the exercise training. All participants recorded individual exercises in the diary, the exercise video was well received and used, and most participants paid attention to the visual rewards. All participants found the technology easy both to learn and to use. The exercise programme adherence was good, with, on average, 77% attendance for the virtual group exercises, and all participants performed additional individual exercises. The average number of physical activity sessions per week was doubled from 2.9 (range 0–10, median 2) at baseline to 5.9 (range 3.3–10.33, median 4.8) during the intervention period.

**Conclusion:**

The results indicate that the tablet-based intervention may be feasible in COPD, and that it was acceptable, encouraged a sense of peer support and fellowship in the group and motivated participants to physical activity and exercise training in daily life. Further assessment is needed on patient outcomes.

## Background

The prevalence of chronic obstructive pulmonary disease (COPD) is increasing, and it is estimated that COPD will become the third leading cause of death globally by 2030 [1]. Physical inactivity is associated with poor health outcomes in COPD [2]**.** It is therefore crucial for patients to have a physically active lifestyle. However, people with COPD are less physically active than healthy people of the same age [3, 4] and than people with chronic bronchitis [5]. In COPD, both physical and behavioural components influence activity levels [2, 6]. Physical activity is defined as “*any bodily movement produced by skeletal muscles that requires energy expenditure”,* while exercise training is *“a subcategory of physical activity that is planned, structured, repetitive”* for fitness purposes [7]*.* Pulmonary rehabilitation, a comprehensive intervention, with exercise training, patient education, and psychosocial support, is vital in the management of COPD [2, 8, 9]. Patient outcomes include increased exercise capacity, improved health-related quality of life (HRQL) and reduced dyspnoea and fatigue [10]. However, it is not clear how to extend the effects of pulmonary rehabilitation, or if the improvements in exercise capacity lead to increased physical activity in everyday life for patients [2].

There is some evidence that behaviour change interventions may increase physical activity in COPD [11]. Home-based interventions for everyday life might be helpful, as travel and transport can be difficult for COPD patients [12, 13]. According to patients, support from peers motivates maintenance of exercise and physical activity in daily life [14, 15]. Technology enables different forms of virtual exercise groups [16], and there are a variety of such technologies, including multiparty videoconferencing, multiplayer exergames, discussion forums and bulletin boards, virtual reality technologies and social networks. Discussion forums and bulletin boards have frequently been used in interventions for physical activity change for healthy or obese populations [17], and online social networks have been used in exercise behaviour interventions for young adult cancer survivors [18] and students [19], among others. Avatars have been used to represent older adults exercising together from home in a virtual gym [20]. In COPD, online videoconference exercise groups have been used in home-based comprehensive pulmonary rehabilitation programmes [21–27] and for group-based exercise training only [28]. Despite the availability of technologies, there are to our knowledge few studies with COPD patients using virtual group technologies for physical activity change or exercise training, besides multiparty videoconferencing. Exceptions include a multifaceted intervention with an online forum on a website [29].

Both endurance and resistance/strength exercise training are important exercise modalities in COPD [2, 30]. Endurance training in the form of ground-based walking requires no exercise equipment; it is easy to incorporate and accessible in everyday life. Ground-based walking both on indoor tracks [31, 32] and outdoors [33, 34] has proved effective in improving patient outcomes. For resistance and strength training, exercise bands might be more accessible in the home setting than conventional equipment such as weight machines. The effects of training with elastic tubing equalled or exceeded those of training with weight machines in terms of various patient outcomes [35]. Merging “*exercise training requirements, with behavioural modification promoting healthy physical activity levels”* is a challenge in COPD [2].

We used an application (app) with functionality for a virtual peer group, a digital exercise diary, a follow-along exercise video, and visual rewards on the home screen wallpaper. The exercise programme combined outdoor ground walking with indoor resistance and strength training, as well as self-chosen exercise training/physical activity. Such a programme could be offered on its own to change physical activity behaviour for COPD patients, as in the present study, or as an exercise maintenance programme after pulmonary rehabilitation.

The aims of this feasibility study were to assess a tablet-based physical activity behavioural intervention in virtual groups for COPD regarding 1) patients’ acceptance 2) technology usability 3) patients’ exercise programme adherence and 4) changes in patients’ physical activity level.

## Method

### The app

#### Virtual peer group

**Fig. 1:**
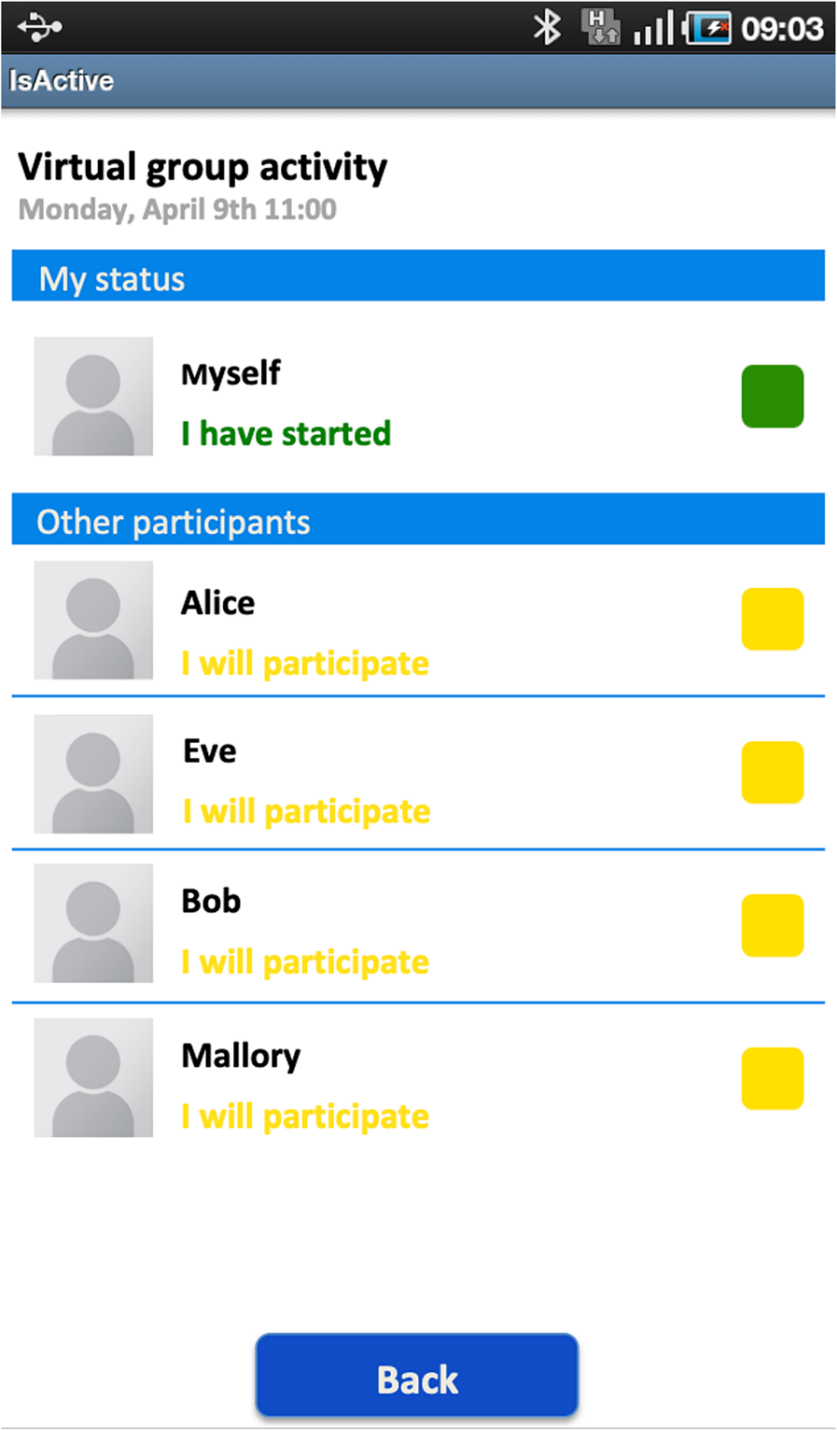
Activity status of the members in a virtual group. Placeholder image by Greasemann [CC BY-SA 4.0], from Wikimedia Commons

The app included functionality for scheduled virtual group exercises, where the participants exercised together in time but not at the same location. For each scheduled virtual group activity, the participant could see the activity status of the other group members. An activity status contained a participant’s thumbnail image, a colour-coded status message and a button (Fig. 1). Participants updated their activity status by pressing the status button to change their status from the default “*I will participate*” (yellow) to either “*I have started*” (light green), “*I have finished*” (dark green), or “*I cannot participate*” (red) (Table 1).

**Table 1:** The predefined text status messages and their corresponding colour code

Text message	Colour
I will participate	Yellow
I have started	Light green
I have finished	Dark green
I cannot participate	Red

### Exercise diary

**Fig. 2:**
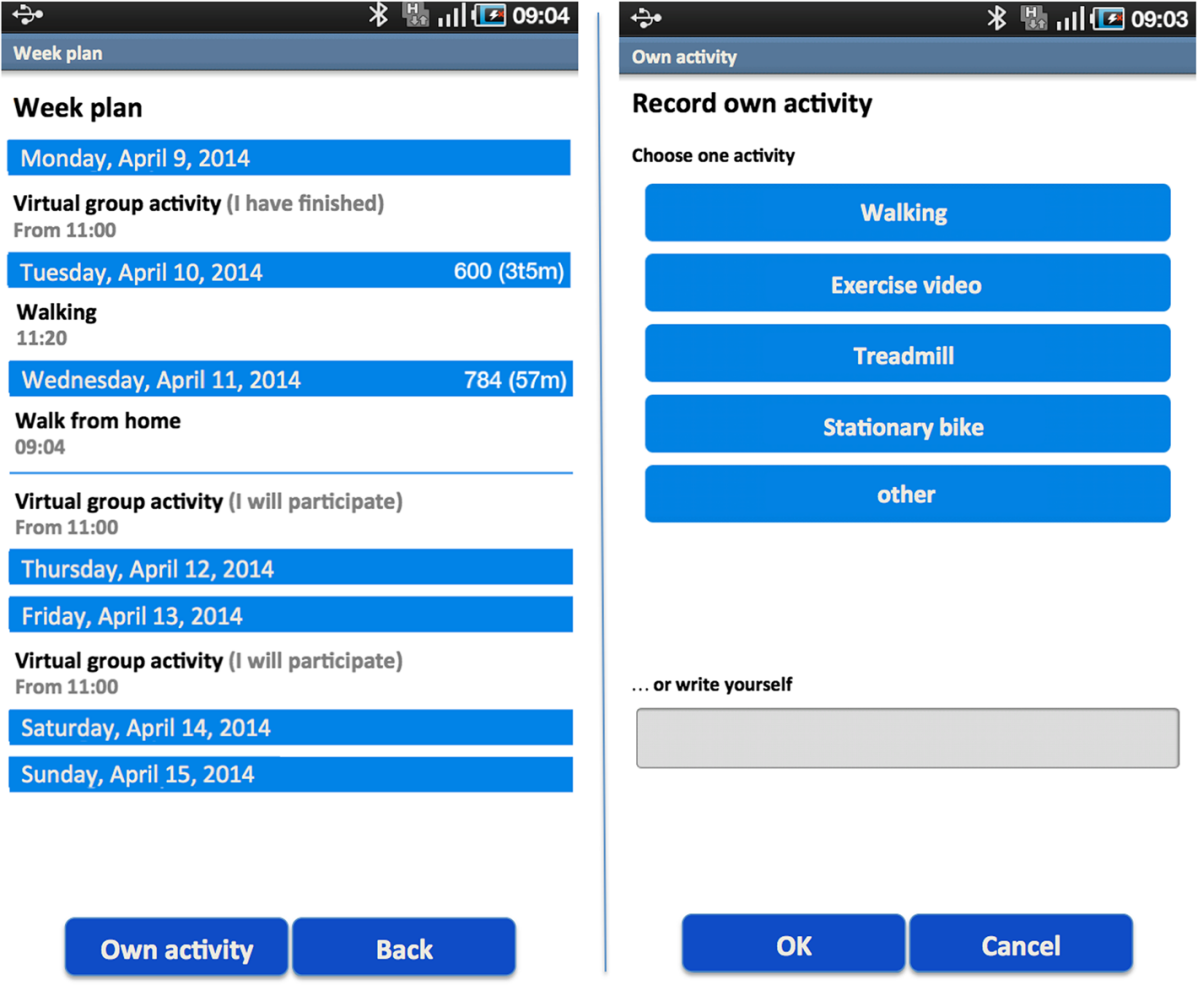
Exercise diary and predefined exercise categories

The app contained an exercise diary providing an overview of planned and attended virtual group exercises for a particular week. A user could also record the individual exercises s/he performed in the diary. The self-recording could be done either by choosing a predefined exercise type (Fig. 2) or by entering a free-text description. Using the latter option, a user could record more detail about an exercise than the standard text option allowed. A user could view his/her historic exercise data (e.g. number of exercises per week). Data from a ProMove-3D activity sensor (Inertia Technology B.V., Enschede, the Netherlands) could also be uploaded to the diary. The sensor provided information about the intensity of a walk (energy expenditure) in the form of Integral of the Modulus of the Accelerometer (IMA) values.

#### Follow-along exercise training video

The app included a follow-along exercise video with resistance and strength exercise training. It lasted 20 min with moderate intensity exercises, combining strength exercises for the lower and upper extremities and thorax stretching, including exercises with elastic bands and weights. The video had a short break halfway, so the user could drink water, etc. The video featured a physiotherapist from a pulmonary rehabilitation clinic. It was shot with a consumer 720p HD video camera at an indoor location with a scenic view, and it had accompanying music.

#### Weather forecast

The tablet had a weather widget on the home screen that visualized the local weather forecast for the next three days (Weather Widgets Yr.no by Pixelspore).

#### Rewards

**Fig. 3:**
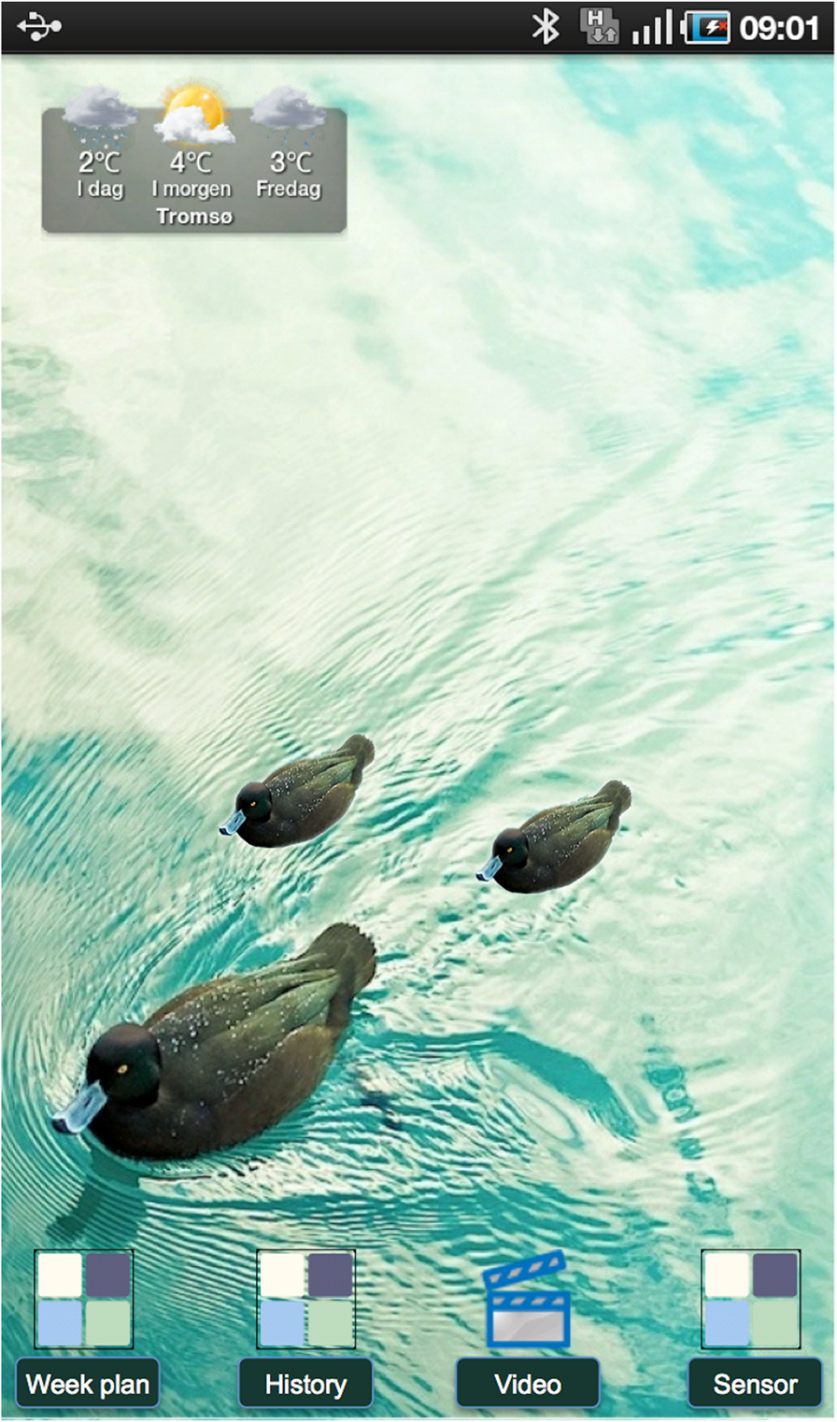
Two rewards on the wallpaper: at the bottom of the home screen there were icons for the exercise diary, the follow-along exercise video and the activity sensor, and in the upper left corner the weather widget. Original wallpaper image from Cuba Gallery, modified image by Cuba Gallery and Lars Vognild [CC BY-SA 4.0]

The home screen wallpaper on the tablet presented non-textual information on a user’s physical activity for a week. A photograph of a single duck in a lake was used as wallpaper (image from Cuba Gallery). During the week, a user would be rewarded with a duckling for each group or individual exercise/physical activity s/he performed (Fig. 3). Rewards are an example of gamification, i.e. the use of game techniques in non-game contexts [36]. The home screen wallpaper was reset to its original form at the start of each week.

### The exercise programme

The participants in an exercise group enrolled at the same time. Three virtual group exercises were scheduled per week for six weeks, walking outdoors twice for a minimum of 15 min with high to moderate intensity and exercising indoors once to the follow-along video. The participants could walk from their own home, from work, or from another place at their convenience, and could use the exercise video where it suited them best. In addition, the participants were to perform as many sessions per week as they could of individual exercise training. The participants were free to choose the type of individual training, as long as it exceeded a minimum of 15 min, and with moderate to high intensity.

The exercise programme commenced with an in-person meeting for each exercise group at the outpatient rehabilitation clinic at the University Hospital of North Norway (UNN). At the meeting, the importance of physical activity for COPD patients was emphasized. The participants were advised on walking speed for health benefits. They were recommended to start fairly slowly and then gradually increase the pace to moderate/high intensity. If the weather was too bad for outdoor walking, the participants were advised to perform an alternative indoor activity, such as ground walking on an indoor track (at home, a shopping mall, etc.), walking on a treadmill, using a step, using the exercise video or a stationary bike, depending on the exercise equipment available and personal preferences. If COPD exacerbations or other medical conditions temporarily prohibited outdoor activities, participants were advised to exercise indoors if possible. A physiotherapist experienced in pulmonary rehabilitation and a lung nurse gave participants advice on how to control their dyspnoea. The group went for an outdoor walk together with the physiotherapist, to become familiar with the suggested walking speed. The participants were provided with sticks, elastic bands and weights to use with the exercise video. The participants agreed on the weekdays and time of day for the virtual group exercises, which had to be performed within an agreed two-hour time slot.

The group of participants received a one-hour training session on how to use the app at the meeting, and they received a user manual with instructions and screenshots. During the programme period, the participants could call technical support if needed. In addition, they were phoned twice by the physiotherapist or a member of the research team to check if any technical problems had been encountered.

The tablet used was a 7-inch Samsung Galaxy Tab P9000 running Android 2.1, where all functionality other than the virtual exercise group app had been disabled or removed. The participants also received a tablet table stand (Samsung Galaxy Tab. 7.0 HDMI Multi-Media Desktop Dock) so it would be easier to view the video during exercise. This would also make the tablet and its home screen wallpaper more visible in the room.

### Behaviour change techniques included

A behaviour change technique (BCT) is defined by Mitchie et al. as [37] “*an observable, replicable, and irreducible component of an intervention designed to alter or redirect causal processes that regulate behaviour; that is, a technique is proposed to be an ‘active ingredient’”*. According to the *BCT Taxonomy v1*, a hierarchical classification system with 93 BCTs divided into 16 clusters [37], the app/exercise programme contained 12 BCTs from nine clusters (Table 2).

**Table 2:** Behaviour change techniques included in the app/exercise programme

Cluster	BCT	App/exercise programme
1. Goals and planning	1.1. Goal-setting behaviour	Goal set to three weekly virtual group exercise sessions, and as many sessions of individual exercises as they could per week in addition. Minimum 15-min sessions, of moderate to high intensity.
	1.2. Problem solving	Advised to perform an alternative indoor activity if the weather was too harsh/medical conditions did not allow outdoor walking.
	1.4. Action planning	Scheduled virtual group exercises. (Frequency, minimum duration, day and time, intensity, type of activity).
2. Feedback and monitoring	2.1. Monitoring of behaviour by others without feedback	The virtual group members see each other’s activity status.
	2.3. Self-monitoring of behaviour	Self-recording of individual exercises in the diary. IMA values. Keeping their virtual group activity status up to date.
4. Shaping knowledge	4.1. Instruction on how to perform a behaviour	The follow-along exercise video. The participants were advised at the start-up meeting on walking speed for health benefits and on how to control their dyspnoea.
5. Natural consequences	5.1. Information about health consequences	Emphasis of the health benefits of a physically active lifestyle at the start-up meeting.
6. Comparison of behaviour	6.1. Demonstration of behaviour	The follow-along exercise video. The outdoor walk with the physiotherapist at the start-up meeting.
7. Associations	7.1. Prompt/cues	Status updates from peers in the virtual group at the time of performance.
9. Comparison of outcomes	9.1. Credible source	At the start-up meeting, a pulmonary rehabilitation physiotherapist and a lung nurse emphasized the importance of physical activity.
10. Rewards and threats	10.3. Non-specific reward	The participants received visual rewards on the wallpaper.
12. Antecedents	12.5. Adding objects to the environment	The tablet on its table stand.

### Study design and ethical and legal issues

This was a feasibility study. Two exercise groups were to be recruited, each with five participants. Due to the weather conditions north of the Arctic Circle, the study was not to be performed mid-winter. The Regional Committee for Medical and Health Research Ethics (REC North) concluded that the study did not need ethical approval. The Data Protection Officer at UNN approved the study. The participants gave written informed consent for participation.

### Data collection and analysis

The empirical sources were semi-structured interviews, the System Usability Scale (SUS), and self-reported data on physical activity. Patients’ acceptance was assessed by the interviews. Acceptance was defined as the extent to which services were generally approved and used, patients’ satisfaction with services and the perceived usefulness of services. Technology usability was assessed by the SUS. Participants’ change in physical activity level was assessed by self-reported data. Exercise programme adherence was assessed in terms of the number of virtual group exercises attended, and whether the participants performed additional individual exercises. The interviews were also used to shed light on specific issues related to technology usability, physical activity and programme adherence.

#### Interviews

The interviews were semi-structured with open questions and follow-up questions. The primary themes of the interviews were user perceptions of the exercise programme, their perceptions and usage of the components of the app, their perceptions on the technical training and learnability of the app and ease of use, perceived benefits/effects of participation, and factors affecting participation. One of the authors, an experienced interviewer, performed the interviews by telephone within a few days post intervention. There were practical and economic reasons for using telephone interviews. The interviewees were fairly geographically dispersed, so we avoided travelling time and cost. Also, the participants were spared having the interviewer visit them in their own homes. However, most important was our positive experience with interviewing COPD patients by telephone [23, 25], where the interviewees were relaxed, they could easy elaborate on difficult themes, and it was possible to elicit high quality data. This is in line with findings by others [38]. The interviews lasted 30–45 min, and were audiotaped and transcribed afterwards, except for one interview where notes had to be taken instead due to technical problems with the recording device. A professional translator translated interview quotes from Norwegian into English.

For the analysis, a theme-centred approach [39] and a “descriptive interpretation” [40] were used. The data were categorized according to the study’s main themes and relevant sub-categories, and positive and negative aspects were extracted in an “issue-focused” [41] cross-case analysis [42]. The data were mainly coded using a deductive approach. Three of the authors analysed the data. The authors performing the interviews and the analysis did not have any previous relationship to the participants, but met the participants at the in-person meeting included in the programme.

#### System usability scale

The SUS is an instrument for subjective assessment of technology usability, and covers aspects such as technical training, complexity and the need for support [43]. It has 10 questions, using a five-point Likert scale, and scores range from 0 to 100, the latter being the best score. An average score above 71.4 indicates good usability, above 85.5 excellent and above 90.9 best imaginable [44]. The SUS was self-administered at home post programme. Average, median and range were calculated.

#### Self-reported data on physical activity

At baseline, the participants reported how many times in a typical week (7-day period) they performed moderate to strenuous physical activities or exercise training with a minimum duration of 15 min. This was done at the in-person meeting. Their physical activity during the programme period was retrieved from the self-reported data in the app’s exercise diary, and average, median and range were calculated. For the difference in physical activity between baseline and the programme period, mean and confidence interval were calculated.

### Patient selection

The inclusion criteria were COPD diagnosis Global Initiative for Chronic Obstructive Lung Disease (GOLD) [45] Grade I - III, previous participation in pulmonary rehabilitation, and living in the vicinity of Tromsø. The exclusion criteria were inability to operate a touchscreen device and difficulties walking outdoors. Healthcare personnel at the outpatient pulmonary rehabilitation clinic at UNN recruited the participants among former rehabilitation attendees during the last few years at the clinic. The recruiters also used their knowledge about eligible candidates’ ability to walk at the time they attended rehabilitation. The participants received both written and oral information about the study.

## Results

### The participants

Ten participants were recruited. One participant was in GOLD grade I, six were in grade II and three in grade III; seven were female and three were male (Table 3). The average age was 65.7 (range 47–74, median 67.5). Nine participants were retired, while one was still working. Three participants were living in a one-person household. All the participants had a mobile phone, which they used for phone and text messages, one of them for receiving text messages only. Two of the participants were using the mobile phone for Internet, four were using the calendar function, one was using alarms and one was using apps on the phone. Eight of the participants were PC users, using a PC several times a week. They were using the PC for Internet, online banking, etc. Three of the participants were using social media. Table 4 shows the kinds of exercise training the participants performed at baseline.

**Table 3:** The participants

Characteristics	*n* = 10	
GOLD Grade		
I	1	
II	6	
III	3	
Gender		
Male	3	
Female	7	
Age		
45–54	1	
`55–64	3	
65–74	6	
Technology use		
Mobile phone	10	
SMS	10	
Internet on the mobile	2	
Computer/Internet	8	
Social media	3	
Computer games	0	
Distance city centre		
< 0.5 km	3	
10–30 km	4	
50–70 km	3	

**Table 4:** Kinds of exercise training at baseline

Kinds of exercise training performed	n = 10
Exercise training at the rehabilitation centre	4
Outdoor walking	4
Swimming	1
Using a step	1
Snow shovelling	2
Exercise programme at home	1

The participants were enrolled in two exercise training groups. The first group (P1-P5) participated from October to November 2011. The second group (P6-P10) participated from April to May 2012. The first group participated in a season with few hours of daylight and harsher weather conditions, while the second group had more stable weather with little snow and more daylight. The participants in the first group exercised once a week at the outpatient rehabilitation clinic together with other COPD patients, while the second group did not. Both groups decided to perform the virtual group exercises around noon. The participant still working had permission from the employer to participate in sessions that took place during working hours. One of the female participants had to withdraw from the study after two weeks due to other illness.

### Acceptance by patients

#### Patient satisfaction and general approval

The participants were satisfied with the tablet-based intervention, as illustrated by the following quotes:

“*It was motivating […] You sort of felt that you had to make a greater effort when you had that [tablet] there, you know. I thought it was great”* P5.

*“It was just the right thing for me. It was a good, gentle kick in the pants”* P6.

*“It’s fantastic motivation for walking, for getting out of the living room, yes, now you need to get over the doorstep and get out.” “I think it was a great programme”* P10.

All of the participants would participate again, as expressed by the following participants:

“*Then I would have said yes in the blink of an eye” P3.*

“*If I had been offered a chance to continue, I would have said yes straight away” “now there’s an empty space […] where it was standing. I would really have liked to put it back again.*” P9.

Further, all participants would recommend others to participate:

“Yes, *Absolutely”* P2.

“*Absolutely, absolutely” “unfortunately there are many people who have a tendency, when they find out that they have COPD, then they say, oh that’s why I’m so short of breath, yes, that’s why I don’t manage to do anything, then I have to sit here - and then it’s good to get a kind of kick in the pants*” P8.

#### Virtual peer group and action planning

All participants except the one who was working updated their own activity status, kept track of the others’ status updates, and perceived the virtual peer group as useful to help them get going, as illustrated by expressions such as:

“*It was quite fun […] to see that some [of the] others had also started, that it wasn’t only me walking”* P3.

*“It is motivating that there are others” “I was curious about [whether] they had got started” P5.*

*“At 10 o’clock, when I switch it on, I see, that one has started now, and then maybe some more […] heavens, here you just have to get going.*” (P7).

*“When you are part of a group, you do feel a bit of an obligation*” P8.

“*I thought, is it motivating in any way to walk together with others in the county that you just log in together with?” “And the motivation, that kick in the pants, it was much stronger than I had expected*” (P9).

The participants found that having a time schedule for the exercises helped them to avoid postponing the exercise training to another time or day:

“*I think that matters a lot. And I think that’s related to getting things in place, otherwise it’s easy to get into that, as most people when they are going to start exercising, yes, no, I had something else I must do today, I’ll do it tomorrow.”* P8.

“*When you don’t have it you can say that no, no, I’ll go tomorrow, you know.”* P10.

#### The follow-along exercise video

The exercise video was well received by the participants, illustrated by expressions such as:

“*Lots of people have seen it, you know […] and thought that it was so well made […] there are actually several people who have asked if they could buy it”* P4.

“*I fell in love with the video, […] that’s what was closest to me’*” P5.

Two participants used the exercise video for the virtual group exercise only, while the others exercised to it several times a week. One participant used another set of exercises that s/he was already using. Almost all the participants placed the tablet in the living room in a place suitable for exercising.

#### Exercise diary

All participants recorded individual exercises in the diary. With one exception, the participants mainly used the predefined text options for describing the type of exercise and entered their own textual description only now and then.

There were technical challenges in integrating the activity sensor with the tablet app, and it was not ready for use by the first group of participants. While a few of the participants in the second group started out using the sensor with the tablet, they stopped after a short period due to technical problems.

#### Rewards

Most of the participants kept track of the number of rewards they achieved during a week, as illustrated by the following expressions:

*“Tried the maximum. Managed it for three weeks, quite fun” P2.*

Responses to the wallpaper were mainly positive, while a few of the participants had no strong opinion about it:

“*It was cute! […] When I walk around [a small lake] […] there are ducks out there […] now I have them at home too*” P3.

The participants had divided opinions about whether they would have preferred seeing the number of rewards the others achieved, as illustrated by the following expressions:

*“No, I’m not kind of a competition person. Not about doing the most, no”* P7.

“*I would have liked to see how active the others had been*” P8.

“*What the others [got], I don’t think that mattered so much*” P10.

#### Problem solving

As recommended, with bad weather or if the participant’s health allowed for indoor but not outdoor activities, the participants performed alternative indoor activities such as stationary biking, walking in a corridor, using a step, or using the exercise video:

“*When the weather was miserable […] I used the exercise bike”* P2.

*“There were actually a couple of times […] when it was so awful outside that I took the walking trip inside back and forth in a long passage”* P6.

“*I had to improvise and do something at home [for health reasons]. I had what they call a mini stepper, I stand and step on that.*” P7.

The weather forecast widget was used by some of the participants, but its usefulness for planning outdoor activities was limited. Many participants continued getting the weather forecast from TV, Teletext, the web, or simply by looking out through the window. In addition, the weather widget stopped working after a few weeks on several of the tablets.

#### Well-being

The participants experienced that participating in the exercise programme influenced their well-being and mood positively. The importance of doing outdoor walking was also emphasized by several of the participants:

*“Speaking for myself, I know that as long as I am out in the fresh air I have a lot of benefit”* P3.

*“You get fitter when you do things outside and you are so satisfied when you come home too, at least I am”* P10.

#### Individual adaptation

The participants took advantage of the opportunity for individual adaptation: one participant went walking with his/her spouse, one went alone or together with his/her spouse, and one participant walked with a neighbour. Another participant exercised to the video with his/her spouse. Some participants used the video frequently for the individual exercising, while others preferred walking or other physical activities**.**

#### Family and friends

Family and friends gave the participants positive feedback:

*“Everyone was very curious about what I had started on now” “it was fine when they heard that it was all about exercising”* P3.

*“There’s been some […] discussion about it, yes” “they have […] been interested”* P4.

“*The kids, they just thought it was fun*” P6.

#### Other aspects

One participant missed the social aspect of exercising physically together with others, while another participant thought that the exercise programme was as good as or better than centre-based rehabilitation programmes. A third mentioned feeling concern that something had happened if someone did not participate in a group session. This concern was due to the severity of COPD.

One participant living in a rural area mentioned the lack of exercise offerings and facilities outside the city centre and the lack of public transportation to the city. A seasonal challenge for outdoor walking, pointed out by another participant living in a rural area, was the darkness and lack of streetlights along the roads, and the lack of footpaths due to deep snow. However, April and May (the light season) was a good period for participation for these reasons.

No differences were found in the participants’ acceptance or physical activity behaviour that could be attributed to the fact that one of the groups also exercised together in person once a week.

### Technology usability

The average SUS score for the participants was 80.5 (range 67.5–95, median 85), indicating good usability of the technology in this context. The interviews also revealed that the participants found the tablet and app easy both to learn and to use, illustrated by expressions such as:

“*I don’t have a computer” “I became a beginner” “I thought it was easy to learn for someone like me”* P5.

The participants found the technical training and the printed user manual sufficient for learning and using the tablet and app. A couple of the participants said they needed some time to get familiar with using a touchscreen. Almost all the participants found the tablet’s 7-inch screen size large enough even for the follow-along exercise video. However, one participant would have preferred a larger screen, while another connected the tablet to the TV while exercising. Two participants experienced a slow network connection a few times; however, this did not seem to influence their acceptance.

### Changes in physical activity level and programme adherence

Physical activity increased for the participants from on average 2.9 (range 0–10, median 2) sessions per week at baseline to 5.9 (range 3.3–10.33, median 4.8) sessions per week during the programme period. Of these, 2.3 (range 1.33–3, median 2.5) were virtual group exercises, and 3.6 (range 1.5–7.3, median 2.3) were individual exercises. This was on average a 77% attendance rate for the virtual group exercise sessions, and all participants performed individual exercises. The difference in physical activity at baseline and during the programme period was on average 3.06 with 95% CI [0.71, 5.40].

The results indicated that there might have been some underreporting of physical activity in the exercise diary:

“*I saw that, that I haven’t recorded as much as I have done, I just haven’t” P4.*

The results also indicated that the walking trips/physical activities tended to exceed the minimum time of 15 min.

## Discussion

### Principal results

The virtual peer group was experienced as motivating, helping participants to get started and be physically active. They updated their own activity status, and kept track of the others’ status. Having a time schedule for the exercises helped them to avoid postponing the exercise training. The wallpaper of the tablet home screen was perceived as positive, and most of the participants paid attention to the number of rewards they received. All participants recorded individual physical exercises in the diary, and the exercise video was well received and used. The participants took advantage of the opportunity for individual adaptation. Preferences varied, ranging from use of the follow-along video for the individual exercises to a stationary bike or a step, while some participants enjoyed outdoor walking the most. Most participants exercised alone, while some also exercised together with a neighbour or partner. Almost all the participants found the 7-inch screen size of the tablet large enough for exercising. Both the average SUS score of 80.5 and the interviews indicated that the participants found the app easy to learn and to use. The programme adherence was good: the attendance of the virtual group exercises was 77%, and in addition all participants performed individual exercise training. The average number of physical activity sessions was doubled from baseline. However, it is not possible to draw any conclusion regarding the significance/clinical relevance of the increase in physical activity behaviour due to the small number of participants.

### Related work

In COPD, there are several studies on technology interventions for physical activity behaviour change for individual patients [34, 46–52]. Many of these include the use of an exercise diary or questionnaire for self-monitoring of behaviour [34, 47, 48, 50], and/or the use of activity sensors and step counters [46, 50, 51]. Single-player exergames have also been used at home for exercising [53], and virtual reality in remotely supervised exercising for COPD [54]. We are not aware of any studies in COPD of physical activity intervention with visual rewards. However, visual rewards are used in such interventions in other populations [55]. Online pulmonary rehabilitation programmes have been delivered to individual COPD patients at home [56] and to online groups of patients [21–27]. However, for COPD, surprisingly few physical activity behaviour change interventions studied include virtual groups, besides videoconferencing exercise groups and an intervention combining an online forum with a pedometer and other functionality [29]. Further, findings by others support the use of multifaceted interventions with the opportunity for individual adaptation for targeting physical activity behaviour change in COPD [57].

### Limitations

The present study was small, and only a first step towards exploring the feasibility of the intervention. As this was not a controlled study, only preliminary results on physical activity level are available, and solid evidence is lacking. The statistical analyses must be interpreted with great caution given the small sample size, which may not be representative of the target population. The positive attitude to outdoor activities in general in Norwegian society might have biased the results and limit generalization. All participants had previously participated in pulmonary rehabilitation, with education covering the health benefits of physical activity in COPD more extensively than the in-person meeting included in the programme allowed for. It is not known whether usage and acceptance would have been different for participants entering the programme without previous rehabilitation experience. For such participants, the health benefits might be less obvious.

The same healthcare personnel who took part in developing and providing the exercise programme recruited the participants, and the participants had previously attended pulmonary rehabilitation/exercise training classes with the physiotherapist who took part in this study. This might have biased the positive results. The exercise video was also with the same physiotherapist. It is not known whether this influenced patient acceptance, or how acceptance would have been if a gym or a patient organization and not a hospital had provided the intervention. It is not known whether the programme sustained long-term physical activity and exercise behaviours, or what programme duration would be optimal.

Physical activity at baseline was reported by subjective means, through recall of a typical week, as in the Godin and Shepard leisure-time physical activity questionnaire (LTEQ) [58]. We did not ask for the number of light exercise sessions, nor did we differentiate between moderate and strenuous exercise. However, for the LTEQ the frequency of moderate and strenuous exercises is suggested as an indicator of the health contribution [58]. The reporting of physical activity during the programme period was also by subjective means, but not through recall of a typical week. Subjective methods lack accuracy when it comes to reporting of duration, frequency and intensity [59]. The intensity of the participants’ walks and other exercise training is unknown. Further, the minimum duration of 15 min per exercise session may not be long enough for health benefits. However, we anticipated that for outdoor walking, for example, once participants had crossed the doorstep, they would be more likely to walk for a longer period than the minimum duration. The results supported this assumption. The exercise video was also of longer duration, lasting 20 min.

It is unknown whether HRQL or exercise capacity improved for the participants, or whether participation influenced self-efficacy. People may refrain from a behaviour due to low self-efficacy, that is, their belief in “*whether they can perform the necessary activities*” [60]. COPD patients’ lack of confidence in their own ability to avoid breathing difficulty while exercising may influence exercise behaviours.

Unfortunately, the existing user acceptance models were not suitable for our study. Both the Technology Acceptance Model (TAM) [61, 62] and its extensions including the Unified Theory of Use and Acceptance [63], for example, have been widely used to predict and explain acceptance. However, as pointed out by Bogazzi [64], neither TAM nor its extensions consider group, cultural and social aspects of technology acceptance, such as “*a personal intention to do something with a group of people or contribute to, or do one’s part of, a group activity*”. Another model, the hedonic-motivation system adoption model [65], does not consider these aspects either.

### Further work

Further work encompasses conducting a randomized controlled trial to assess whether the tablet-based intervention increases physical activity in daily life for COPD patients, and if it could be used for long-term maintenance of patient outcomes after pulmonary rehabilitation. The optimal programme duration also needs further investigation.

## Conclusions

The results indicate that the tablet-based intervention may be feasible in COPD, and that it was acceptable, encouraged a sense of peer support and fellowship in the group and provided motivation for increased physical activity in daily life. Further assessment on patient outcomes is needed.

## Abbreviations

BCT: Behaviour Change Technique
COPD: Chronic Obstructive Pulmonary Disease
GOLD: Global Initiative for Chronic Obstructive Lung Disease
HRQL: Health-Related Quality of Life
IMA: Integral of the Modulus of the Accelerometer
LTEQ: Leisure-time Physical Activity Questionnaire
SUS: System Usability Scale
TAM: Technology Acceptance Model
UNN: University Hospital of North Norway
